# Radiographic features in 2D imaging as predictors for justified CBCT examinations of canine-induced root resorption

**DOI:** 10.1259/dmfr.20210165

**Published:** 2022-01-01

**Authors:** Amanda K.H. Andresen, Malin V. Jonsson, Gerhard Sulo, Dorina S. Thelen, Xie-Qi Shi

**Affiliations:** 1 Department of Clinical Dentistry, Section for Oral and Maxillofacial Radiology, University of Bergen, Norway, United Kingdom; 2 Oral Health Centre of Expertise in Western Norway, Bergen, Norway, United Kingdom; 3 Centre for Disease Burden, Division of Mental and Physical Health, Norwegian Institute of Public Health, Norway, United Kingdom; 4 Department of Oral Maxillofacial Radiology, Faculty of Odontology, Malmö University, Malmö, Sweden

**Keywords:** Cone-beam computed tomography, radiation effects, tooth, impacted

## Abstract

**Objectives:**

This retrospective observational study aimed to evaluate the diagnostic accuracy of two-dimensional radiographs on canine-induced root resorption (CIRR) in lateral incisors and identify predictors of CIRR in patients with impacted maxillary canines (IMC).

**Methods:**

Ninety-nine patients aged 9–17 years, with 156 IMCs, were included in the study. All had CBCT-volumes and two-dimensional radiographs consisting of at least one panoramic radiograph. Two radiologists jointly viewed all cases twice. First, radiographic features related to the IMC and possible CIRR were recorded from two-dimensional radiographs. Then, CIRR was determined from CBCT and according to position and extension classified as mild, moderate and severe.

**Results:**

CIRRs was detected in 80% of lateral incisors (mild: 45%; moderate: 44%; severe: 11%). The sensitivity was generally low at mild and moderate cut-offs (29 and 29%), and somewhat higher for severe (50%). Corresponding specificities were 48%, 63% and 68%. Canine cusp-tip superimposing the lateral incisor’s middle third and root/crown ratio >1 was positively associated with mild CIRR, with an odds ratio (OR) of 3.8 and 6.7, respectively. In addition, the root development stage was positively associated with moderate/severe CIRR when the canine root was nearly or fully developed (OR = 3.1).

**Conclusions:**

The diagnostic accuracy of two-dimensional radiographs was inadequate for detecting CIRR amongst patients referred for CBCT examinations. Based on our results, none of the suggested two-dimensional radiographic features could predict moderate/severe CIRR except for root development stage. IMC in a later stage of root development seems to be associated with a higher risk of moderate/severe CIRR.

## Introduction

Root resorption is defined as a loss of mineralised substance due to the differentiation and activation of multinucleated giant cells called odontoclasts.^1^ Different signalling pathways have been suggested to understand the mechanism of physiological resorption of primary teeth and pathological root resorption in the permanent dentition.^2^ A subtype of external root resorption, such as canine-induced root resorption (CIRR), is pressure-dependent and often induced by adjacent ectopic or impacted teeth.^3^ This pressure-dependent process is described as originating from an eruption-related trauma caused by the canine cusp-tip to the protective pre-cementum layer of an adjacent root surface. If the pressure is not relieved, the resorptive process may invade the underlying cementum as well as dentine.^4^ CIRR is a common sequela of impacted maxillary canines, with an incidence of up to 89.61%, depending on the population examined and radiographic modality applied.^5^ Intraoral periapical or occlusal radiographs, or panoramic radiographs (PAN) and lateral cephalograms are the available two-dimensional modalities for assessing impacted maxillary canines and diagnosing CIRR. However, the diagnostic accuracy of two-dimensional radiographs may be questionable due to the superimposition of anatomical structures, distortion and magnification.^6^

In the past two decades, the application of volumetric scans in dentistry has increased dramatically due to the introduction of cone-beam computed tomography (CBCT). CBCT utilises a cone-shaped X-ray beam to directly create volumetric data of a predefined field of view.^7,8^ Evidence-based guidelines on the use of CBCT state that CBCT may be indicated to assess the localisation of impacted teeth as well as identify possible root resorption when the information cannot be obtained adequately by lower dose two-dimensional radiography.^9–11^ Assessment of possible CIRR was the most common referral question for a CBCT examination of children and adolescents in Sweden.^12^ Several *ex-vivo* studies have pointed to the superiority of CBCT in accurately diagnosing and assessing both simulated and external root resorption compared to two-dimensional radiographs, especially concerning mild and moderate resorption in the apical third of the affected root.^13–15^ Furthermore, CBCT increases the detection rate of CIRR by up to 63% when compared to two-dimensional radiographs.^16^

Radiation protection is an important issue to consider, especially in the younger population, since their risk for stochastic effects is three times higher than adults.^9^ Kadesjö et al^17^ estimated that the radiation burden of a CBCT was between 15 and 132 times that of conventional radiographic examinations when examining the anterior maxillary region in children. Thus, appropriate justification of CBCT in addition to two-dimensional radiography is important to keep the radiation burden as low as possibly achievable for this young group of patients. In addition, one should ask if the exact knowledge of root resorption is crucial for further therapeutic planning and whether all CIRR impact the long-term viability of the affected tooth when recommending a diagnostic modality applying ionising radiation.^18^

Pressure-dependent root resorption is a possible sequela of impacted teeth which often goes unnoticed without subjective symptoms and major consequences. Therefore, CBCT examination to determine the precise position and extension of a CIRR is only justified in selected cases where the presence of a CIRR could potentially alter the preliminary treatment plan. A typical treatment plan could, for example, be surgical exposure of the impacted canine and extraction of a premolar followed by space closure; in this context, information about possible severe root resorption on an adjacent lateral incisor is crucial for the choice of extraction, thus avoiding extracting a healthy in favour of a severely resorbed one.^19^ Due to the cementum’s ability to self-repair, the loss of cementum may not be permanent.^20^ In addition, pressure-dependent CIRR does not induce pathological mobility as long as the remaining root length is longer than 1 cm.^21,22^ Hence, CIRR confined to the cementum or localised within the incisor’s apical third may not be sound criteria for justifying a CBCT examination because the long-term functionality in terms of tooth stability is uninfluenced. In this study, we aimed to evaluate the diagnostic accuracy of two-dimensional radiographs to detect CIRR in lateral incisors and identify predictors of CIRR in patients with impacted maxillary canines for justified CBCT examinations.

## Methods and materials

### Study design and patients

**Figure 1.**
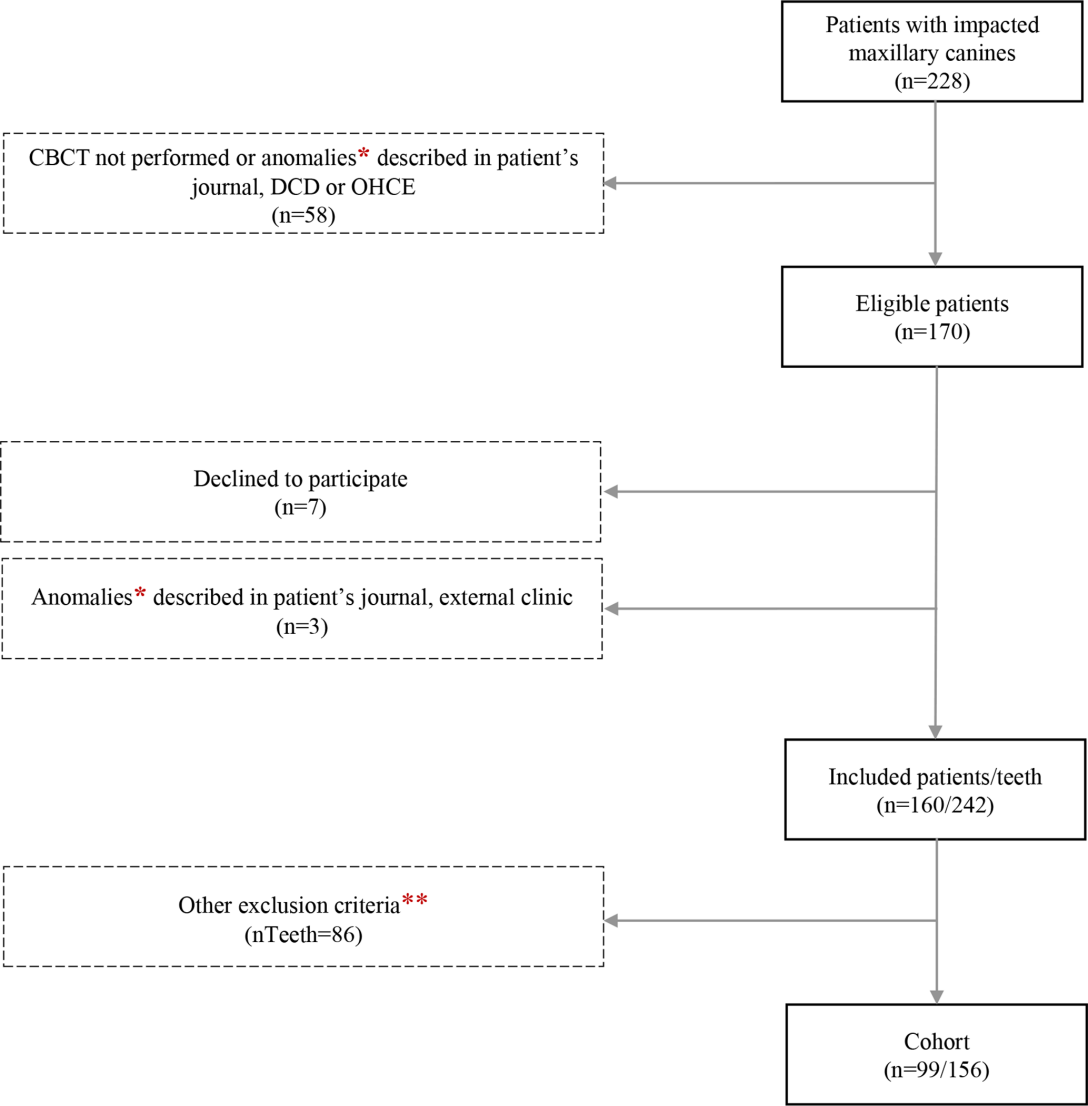
Process of inclusion and exclusion of patients for the study population. * Lip-jaw-palate clefts/syndromes with craniofacial abnormalities** ≥18 years; no panoramic radiograph within 6 months of cone-beam CT examination; previous traumatic dental injuries towards the anterior maxilla; previous or ongoing orthodontic traction of the impacted maxillary canine; maxillary lateral incisor agenesis; detected canine-induced root resorption of only central incisor or first premolar on cone-beam CT.

Among all performed CBCT examinations at either the Department of Clinical Dentistry (DCD), University of Bergen, Norway, or Oral Health Centre of Expertise in Western Norway (OHCE) between January 1, 2013, and October 30, 2017, 228 patients were identified with referral questions of suspected CIRR and/or ectopically positioned unerupted maxillary canine(s). Pre-selection of eligible patients was conducted by reviewing electronic patients’ journals; 58 cases were excluded due to CBCT examination not carried out or orofacial abnormalities. An invitation letter was sent to 170 patients with a prepaid reply slip to be returned when the patients or proxy wished to decline participation in the study. Seven patients declined participation, and another three were excluded after data collection, owing to orofacial abnormalities described in electronic patients’ journals at external clinics (Figure 1).

At the image assessment stage, the inclusion criteria were patients aged 9–17 years at the time of referral, with at least one available PAN obtained within six months apart from the CBCT examination. The amount and type of additional two-dimensional radiographs varied for the individual patient and were most commonly periapical intraoral radiographs and/or horizontal tube-shift intraoral radiographs, and for some, cephalograms. Exclusion criteria were previous traumatic dental injuries towards the anterior maxilla, orthodontic traction of the impacted maxillary canine (IMC) and agenesis of the maxillary lateral incisor. Cases with CIRRs that did not concern the maxillary lateral incisor were also excluded after the radiographic assessment. These exclusions were not mutually exclusive, resulting in a cohort of 99 individuals with 156 IMCs (Figure 1).

### Data collection

Relevant digital radiographs, as well as information on patients’ age, sex, uni- or bilateral IMCs, traumatic dental injuries, orthodontic traction of the IMC, maxillary lateral incisor agenesis and time interval between two-dimensional radiographic and CBCT examination, were collected from patients’ journals at DCD, OHCE and/or external dental clinics.

The CBCT machines used included Accuitomo-170 at DCD (J. Morita MFG. CORP, Tokyo, Japan) and Veraviewepocs 3D R100 at OHCE (J. Morita MFG. CORP, Tokyo, Japan). The exposure parameters varied depending on the optimisation and individual adjustment of exposure parameters and the applied machine. For Veraviewpocs 3D R100, the exposure parameters were 80–90 kV, 5–10 mA and 9.3 or 9.4 s, with a field of view of 4 × 4 cm in all but one case, where it was 8 × 8 cm. The voxel size was 0.125 mm in all cases. The corresponding exposure parameters for 3D Accuitomo-170 were 80–90 kV, 5.5–10 mA,and 5,4, 9.4 or 17.5 s, with a field of view of either 4 × 4 cm or 6 × 6 cm, yielding a voxel size of 0.08 mm or 0.125 mm, respectively.

### Radiographic assessment

Two experienced maxillofacial radiologists jointly viewed the radiographs for all cases in two sessions with a time-lag of nine weeks; consensus was reached for all cases. Material for the first session consisted of two-dimensional radiographs utilising Planmeca Romexis viewing software (Planmeca, Helsinki, Finland). The second session consisted of CBCT volumes, and OneVolumeViewer was utilised (J. Morita MFG. CORP, Tokyo, Japan). Viewing took place in a quiet room with dimmed light. Cases were viewed in a randomised sequence. The radiologists were blinded at the patient level. The only information available was regarding uni- or bilateral impactions. Two monitors (Fujitsu P27-8 Te Pro) were pre-adjusted according to DICOM settings with a resolution of 2560 × 1440. The observers were allowed to perform image enhancement according to their preference.

Radiographic features (predictor variables) recorded on PAN included peg-shaped lateral incisor within the same quadrant, distal tipping of lateral incisor, the position of canine cusp-tip to the lateral and central incisors’ roots, root development of the IMC, and inclination of the central axis of the IMC in regard to the midline, central axis of the lateral incisor and secant line of the occlusal plane in the antagonising quadrant. Detailed information on the applied criteria for registration of radiographic variables can be found in the Supplementary Material 1. In addition, CIRR in the lateral incisor based on two-dimensional radiographs were assessed using a four-step confidence scale, namely, CIRR definitely present, CIRR probably present, CIRR probably not present and CIRR definitely not present. Assessment of CIRR in the lateral incisor on CBCT volumes was considered the gold standard (outcome variable) and recorded as dichotomous data. In the presence of root resorption, the position of the resorption lacunae was defined as localised in the root’s apical 1/3, middle 1/3, cervical 1/3 or a combination of the above mentioned. In contrast, the extension of the resorption lacunae was classified as limited within cementum layer (defined as slight root surface flattening), outer half of dentine, inner half of dentine or pulp involvement. Based on the resorption lacunaes’ position and extension, we classified CIRR into mild, moderate and severe, considering the self-repair capacity and the impact of substance loss on the future stability of the resorbed tooth. Thus, CIRR confined to the cementum or at the apical 1/3 of the lateral incisor’s root on CBCT volumes were classified as mild, whereas CIRR extending to the inner half of dentine and localised at the middle and coronal part of the root were severe and the remaining CIRR were classified as moderate.

Supplementary Material 1.

### Statistical analyses

Analyses regarding CIRR were conducted at the tooth level. Continuous variables are presented as mean and standard deviation (SD) or median and interquartile range (IQR), whereas categorical variables are presented as proportions. The diagnostic efficacy of two-dimensional radiographs on the assessment CIRR at cut-offs of mild, moderate and severe was calculated in terms of sensitivity and specificity. “Definite” and “probable” CIRR were defined as CIRR, and “not-probable” and “definitely no” were defined as resorption free when calculating the sensitivity and specificity. Univariate logistic regression analysis with clustered robust standard errors accounting for the clustering of observations was used to identify possible associations between radiographic features registered on PAN with CIRR diagnosed on CBCT. We conducted univariate logistic regression analysis with the presence of mild, moderate and severe CIRRs as outcomes. Results are expressed as odds ratio (OR) and their corresponding 95% confidence intervals (CI).

### Ethical considerations

Ethical approval for conducting this study was obtained by the Regional Ethics Committee in Western Norway (REC2017/286). All included patients gave passive consent. Consented inclusion refers to the acceptance of the retrospective material collection, with no impact on the diagnosis or treatment of the eligible patient. All material was de-identified upon registration and stored on a password-protected encrypted hard drive.

## Results

### Included patients and their characteristics

We included 99 individuals in the analyses, of whom 57 were female (Table 1). Sixty-eight patients had bilateral impactions; amongst these, only one of the IMCs was included from 11 patients. Thus, the total number of IMCs analysed was 156 teeth. Of the examined IMCs, 48.1% were located on the patients’ right side, and 51.9% were located on the left side. A peg-shaped lateral incisor in the same quadrant as the IMC was present in 12.8% of cases, and 52.6% of the lateral incisors were distally tipped. The median angulation (interquartile range) between the IMC’s central axis and midline was 26.7 (21.2) degrees. The corresponding angulations between the IMC’s central axis and occlusal plane and lateral central axis were 57.6 (18.0) and 33.6 (21.0) degrees, respectively. In 30 (19.2%) cases, the IMC had a fully developed root with a closed apex. The majority of IMCs superimposed on the incisors’ roots (65.4%), most often localised within the middle third of the lateral incisor’s root (23.7%). In 11.5% of cases, the canine cusp-tip overlapped even the central incisor. The IMCs that did not overlap with the adjacent teeth were referred for CBCT examination either prior to surgical removal of the IMC or due to suspicion of ankylosis.

**Table 1.** Characteristics of all predictive variables, including patients/teeth and radiographic features

Cohort characteristics	Total (*n* = 156)	Male (*n* = 66)	Female (*n* = 90)
No. of individuals	99	42	57
Age (in years), mean (SD)	11.2 (1.6)	11.4 (1.8)	11.0 (1.5)
Canine involved			
13	75 (48.1)	30 (45.5)	45 (50.0)
23	81 (51.9)	36 (54.5)	45 (50.0)
Position of CCT, n (%)			
Free of overlapping	54 (34.6)	28 (42.4)	26 (28.9)
Overlapping with lateral incisor on the apical 1/3	15 (9.6)	7 (10.6)	8 (8.9)
Overlapping with both lateral and central incisors on the apical 1/3	1 (0.6)	0 (0)	1 (1.1)
Overlapping with lateral incisor on the middle 1/3	37 (23.7)	10 (15.2)	27 (30.0)
Overlapping with both lateral and central incisors on the middle 1/3	2 (1.3)	1 (1.5)	1 (1.1)
Overlapping with lateral incisor on the cervical and inferior of cervical 1/3	32 (20.5)	17 (25.8)	15 (16.7)
Overlapping with both lateral and central on the cervical and inferior of cervical 1/3	15 (9.6)	3 (4.6)	12 (13.3)
Angulation, midline (in degrees), median (IQR)	26.7 (21.2)	24.5 (15.0)	28.9 (22.2)
Angulation, occlusal plane (in degrees), median (IQR range)	57.6 (18.0)	58.7 (17.0)	54.5 (19.4)
Angulation, lateral incisor (in degrees), median (IQR range)	33.6 (21.0)	31.2 (19.5)	35.5 (22.3)
Root development, n (%)			
Root shorter than crown	9 (5.8)	6 (9.1)	3 (3.3)
Root longer than crown	66 (42.3)	31 (47.0)	35 (38.9)
Close to full length with open apex	50 (32.1)	16 (24.2)	34 (37.8)
Full development with closed apex	30 (19.2)	12 (18.2)	18 (20.0)
Uncertain	1 (0.6)	1 (1.5)	0 (0.0)
Peg-shaped lateral incisor, n (%)	20 (12.8)	6 (9.1)	14 (15.6)
Distally tipped lateral incisor, n (%)	82 (52.6)	35 (53.0)	47 (52.2)
Time lag from PAN to CBCT, n (%)			
≤1 month	57 (36.5)	23 (34.8)	34 (37.8)
1–3 months	78 (50.0)	31 (47.0)	47 (52.2)
3–6 months	21 (13.5)	12 (18.2)	9 (10.0)

CBCT: Cone-Beam Computed Tomography;CCT: Canine cusp-tip; IQR: Interquartile range; PAN: Panoramic radiograph.

Of all the 156 lateral incisors, CIRR was detected in 125 (Table 2). The majority of CIRRs were positioned at the middle third of the roots (*n* = 69), and most often extending into the outer half of dentine (*n* = 67). The middle third of the root and outer half of the dentine was also the most prevalent combination of position and extension (*n* = 39). Ten CIRRs extended into the pulp, of which three were localised at the apical third of the root. According to our classification criteria, 56 (44.8%) had mild CIRR, 55 (44.0%) had moderate CIRR, and 14 (11.2%) had severe CIRR (Table 2). Table 3 shows the distribution of canine crown position in relation to the presence of CIRR according to CBCT assessment. One-hundred and four impacted canines (66.7%) were buccally positioned, while 36 (23%) were positioned palatally. However, the CIRR rates were comparable (82.7% vs  88.9%) regardless of canine crown position.

**Table 2.** Distribution of position and extension of the resorptions according to cone-beam CT assessments. Root resorptions were classified into mild (*n* = 56), moderate (*n* = 55) and severe (*n* = 14), as marked in light grey, grey and dark grey

Extension	Position				
	Apical 1/3	Middle 1/3	Cervical 1/3	Combination	Total
Cementum layer	9	25	3	2	39
Outer half of dentine	12	39	5	11	67
Inner half of dentine	2	5	-	2	9
Pulp involvement	3	-	-	7	10
Total	26	69	8	22	125

**Table 3.** Distribution of canine crown position and presence of resorption according to CBCT assessments

Canine crown position	Canine-induced root resorption		
	Yes	No	Total
Buccal	86 (82.7%)	18 (17.3%)	104
Palatal	32 (88.9%)	4 (11.1%)	36
Midalveolar	7 (58.3%)	5 (41.7%)	12
Not relevant	0 (0.0%)	4 (100%)	4
Total	125 (80.1%)	31 (19.9%)	156

### Diagnostic accuracy of two-dimensional radiographs

In addition to PAN, 82.7% of cases had available periapical intraoral radiographs, of which 51.9% of the cases also had horizontal tube-shift intraoral radiographs. When considering the diagnostic accuracy at the cut-off level of mild CIRR, the sensitivity and specificity were 28.8 and 48.4% for the two-dimensional radiographs. The corresponding sensitivity and specificity were 29.0 and 63.2% at cut-offs of moderate CIRR and 50.0 and 68.3% at cut-off of severe CIRR (Table 4).

**Table 4.** The diagnostic accuracy of conventional radiographic examination on detecting Canine-induced root resorption. CBCT: cone-beam computed tomography

	CBCT examination					
	Cutoff: mild		Cutoff: moderate		Cutoff: Severe	
	Resorption	No resorption	Resorption	No resorption	Resorption	No resorption
Conventional radiographic examination						
Resorption	36	16	20	32	7	45
No resorption	89	15	49	55	7	97
Sensitivity	36/ (36 + 89)=28.8%		20/ (20 + 49)=29.0%		7 (7 + 7)=50.0%	
Specificity	15/ (15 + 16)=48.4%		55/ (55 + 32)=63.2%		97/ (97 + 45)=68.3%	

### Odds ratios associated with findings on two-dimensional radiographs

Due to the low number of severe CIRR, 14 (8.9%), the outcomes variables were only defined at the cutoff for mild-CIRR (set one) and moderate CIRR (set two) for the univariate logistic regression analysis. Table 5 summarises the odds ratios of CIRR confirmed in CBCT volumes associated with relevant radiographic features on PAN. In the analyses involving mild CIRR (set one, Table 5), superimposition of the canine cusp-tip within the middle third of the lateral incisor’s root was associated with a 3.8-fold increase in odds (95% CI: 1.2–12.4) of being diagnosed with CIRR in CBCT volumes, compared to IMCs with a canine cusp-tip free of overlapping. Furthermore, IMCs with ongoing root development and a root/crown ratio >1 was associated with a 6.7-fold increase in odds (95% CI: 1.7–31.2) of CIRR compared to IMC with a root/crown ratio <1. Regarding moderate/severe CIRR (set two, Table 5), root development close to full root length with an open apex and fully developed IMC with a closed apex was associated with a 3.1-fold increase in odds (95% CI: 1.4–36.7) compared to IMCs with root length shorter than crown length.

**Table 5.** Association between age, sex and features on panoramic radiographs, and canine-induced root resorption assessed on cone-beam computed tomography

Variables	OR (95% CI)	
	Set one	Set two
Sex		
Female	1^ref^	
Male	0.86 (0.39–1.91)	0.71 (0.37–1.35)
Age (per one year increase)	0.84 (0.67–1.06)	0.96 (0.79–1.17)
Position of CCT		
Free of overlapping	1^ref^	1^ref^
Overlapping with lateral incisor on the apical third	2.99 (0.61–14.73)	0.79 (0.24–2.72)
Overlapping with lateral incisor on the middle third	3.79 (1.16–12.41)	1.07 (0.46–2.52)
Overlapping with lateral incisor on the cervical third or inferior of cervical third	1.99 (0.69–5.73)	2.02 (0.83–4.91)
Overlapping with lateral and central incisor on the cervical third or inferior of the cervical third	2.99 (0.61–14.73)	2.36 (0.73–7.59)
Angulation, midline plane		
0–30 degrees	1^ref^	1^ref^
31 + degrees	0.62 (0.28–1.37)	0.80 (0.42–1.54)
Angulation, occlusal plane		
0–60 degrees	1.02 (0.46–2.26)	1.35 (0.71–2.56)
61 + degrees	1^ref^	1^ref^
Angulation, lateral plane		
0–30 degrees	1^ref^	1^ref^
31 + degrees	1.12 (0.51–2.47)	1.47 (0.77–2.81)
Root development		
Root shorter than crown	1^ref^	1^ref^
Root longer than crown	6.74 (1.46–31.16)	1.09 (0.57–2.10)
Close to full root length with open apex and full development with closed apex	2.57 (0.63–10.54)	3.13 (1.39–36.67)
Peg-shaped lateral incisor		
No	1^ref^	1^ref^
Yes	0.53 (0.18–1.50)	0.64 (0.24–1.71)
Distally tipped lateral incisor		
No	1^ref^	1^ref^
Yes	1.71 (0.77–3.78)	1.48 (0.78–2.79)

CCT: Canine cusp-tip; CI: Confidence interval;OR: Odds ratio.

## Discussion

In clinical practice in the Nordic countries, periapical and occlusal intraoral radiographs and the horizontal tube-shift techniques are commonly used as the initial radiographic assessment of IMCs. In many cases, two-dimensional radiography is sufficient to obtain necessary diagnostic information for appropriate management of the IMCs. Over the recent years, CBCT has been recognised as a superior tool in diagnostics and treatment planning of IMCs. However, CBCT entails a risk to the patient due to a higher radiation dose than two-dimensional radiographic examinations with PAN and intraoral radiograph.^17^ Previously investigated low-dose protocols have been shown to effectively reduce patient dose while maintaining sufficient image quality for assessing the enamel-dentine junction and distinguishing between lamina dura and periodontal ligament space.^23,24^ Nevertheless, the effective doses of CBCT remain higher than two-dimensional radiography, while the magnitude of the difference between modalities decreases to a certain extent.^23,24^ When evaluating whether CBCT is indicated due to suspected CIRR, both the position and extension of root resorption should be taken into consideration since mild CIRRs may not have a clinical impact on the long-term stability of the resorbed tooth once the pressure from the canine is released. Therefore, justified use of CBCT according to radiographic predictors on PAN for moderate/severe CIRR may serve as a practical approach in radiation protection for children with IMCs, which has not yet been investigated.

Among the investigated radiographic features on PAN, we found that only the IMC’s root development stage was significantly associated with moderate/severe CIRR; the odds increased when the root was nearly or fully developed. This finding is reasonable since an IMC with nearly or fully developed root represents a later eruption stage compared to an IMC with a root length shorter than its crown. Consequently, it has a higher probability of closer proximity between the IMC and lateral incisor’s root, and thus a higher risk of CIRR. We had expected that the position of canine cusp-tip would have predicted a significantly decreased odds of moderate/severe CIRR when overlapping was limited within the apical 1/3 of adjacent roots. However, this could not be verified in the current study, most likely due to the projection characteristics of PAN technique, that is*,* the actual contact point between the canine cusp-tip and incisor’s root for a palatally positioned IMC will be projected towards the apex. In addition, due to PAN technique’s tomographic nature, cautions need to be taken when interpreting radiographic features, such as angulation and root development stage. Particularly if the apex is displaced buccally or palatially. Furthermore, most patients in our sample had less than 3 months between PAN and CBCT examinations. However, 21 (13.5%) had up to 6 month time lag, which may also contribute to an altered overlapping position between canine and lateral incisor.

Regarding other radiographic features on PAN and cohort characteristics that can predict mild CIRR, the present study was unable to demonstrate an association between CIRR and age, sex, measurements of angulations, presence of a peg-shaped lateral incisor or distal tipping of the lateral incisor. Such predictors have previously been reported by Brin et al.,^25^ Lai et al.,^26^ Chaushu et al.,^27^ Alqerban et al^28^ and Guarnieri et al.^29^ However, regarding the position of the canine cusp-tip, our findings comply with numerous studies.^29–33^ Yan et al^34^ found close physical proximity between the IMC and neighbouring root and a fully developed root to be the most important predictors of CIRR. Radiographic features on PAN that predict mild CIRR should not be applied as indicators for CBCT examination. However, knowing the risk factors of mild CIRR may assist orthodontists on whether and how to move the impacted canine away from the tooth with suspected CIRR.

In terms of sensitivity and specificity of the two-dimensional radiographs in detection and assessment of CIRR, we found the diagnostic accuracy inadequate for mild and moderate CIRR. For severe CIRR, the diagnostic accuracy was somewhat higher; about 2/3 of “sound roots” were correctly identified as non-resorbed by conventional radiographs. However, half of the severe CIRR still went undetected. The consequence of missing a severe CIRR may be an incorrect decision of extraction strategy, inefficient use of mechanics and vectors during orthodontic treatment, a questionable periodontal prognosis, or even costly prosthetic treatment later on in some cases. However, these findings cannot be extrapolated and used in an orthodontic practice since the patients in our study were those referred to CBCT examination as findings in two-dimensional radiographs were deemed insufficient.

The inadequate diagnostic performance of two-dimensional imaging does not imply that CBCT shall be taken for all cases when IMC and the neighbouring incisors approximal space cannot be freely projected by two-dimensional radiographs. The patient group in our study represents only a small fraction of all IMCs: the cases deemed as more difficult after two-dimensional examination. In addition, intraoral radiographs were not available for all patients since they were retrospectively collected from the referral clinics. Consequently, the diagnostic accuracy of two-dimensional radiography may be underestimated. To justify a CBCT examination, the suspected moderate/severe CIRR shall also be the crucial diagnostic information for the orthodontic treatment plan. Previous studies reported that treatment plans were altered after CBCT in less than 30% of the impacted canines.^35–37^ Hence, in clinical situations, CBCT is indicated when two-dimensional radiographs could not rule out moderate or severe CIRR, provided the presence of CIRR is essential for treatment choice.

The present investigation assessed radiographic features on PAN that can predict CIRR and that might indicate CBCT examination in patients with suspected moderate/severe CIRR. The concept of moderate/severe CIRR is based on the future functionality in terms of tooth stability of the lateral incisor. According to the efficiency ladder of a diagnostic method introduced by Fryback and Thornbury,^18^ this study lies on the diagnostic impact level. The justification process before a CBCT examination may be further enhanced by studying treatment relevant CIRR. If the presence of moderate/severe CIRR does not influence the follow-up orthodontic treatment, CBCT is not justified. Identification of CIRR is a major benefit of CBCT, which has an essential diagnostic impact on extraction strategy in treatment planning. For facilitating the necessity of other diagnostic information in the planning of surgical exposure or orthodontic traction, vectors needs to be further investigated through multidisciplinary collaboration among dentomaxillofacial radiology, orthodontics and paediatric dentistry.

There are several limitations of the current study that are related to the retrospective study design. The image quality of two-dimensional radiographs could not be standardised since most of the radiographs were collected from the referral dental clinics. Also, CBCT images were obtained using two CBCT devices. The possible impact of examination protocols on diagnostic accuracy of CIRR could not be verified. In addition, the lack of records of possible interceptive treatment between two-dimensional and CBCT examination may also affect the interpretation of the findings.

In comparison with similar CT or CBCT studies, our results display a prevalence of CIRR amongst the highest reported in similar cohorts.^5,38^ Contributing to this are two likely causes, namely the cohort and the applied classification criteria of CIRR. The majority of patients in this study were referred to CBCT examination due to suspicion of CIRR; the prevalence of CIRR may thus be higher than patients with IMCs in general. Also, there is no consensus in the literature regarding the classification criteria of pressure-dependent RR. Ericson and Kurol^39^ were the first to present a CIRR-classification based on CT volumes and *ex-vivo* examinations after extraction. They defined severe CIRR as those involving the pulp, regardless of position. Falahat et al^40^ and Kim et al^41^ also defined severity depending on the extension and omitted assessment of the position. Chaushu et al,^27^ on the other hand, defined severe CIRR as those covering more than one-third of the root length, omitting assessment of extension towards the pulp. Ucar et al^42^ looked at the root volumes of the lateral incisors to diagnose resorption. We classified the severity of CIRR into three categories considering both position and extension of the external root resorption. Further work is required to validate the clinical relevance of the suggested classification criteria.

## Conclusions

The diagnostic accuracy of two-dimensional radiographs was inadequate for detecting CIRR amongst patients referred for CBCT examinations. Based on our material, none of the suggested two-dimensional radiographic predictors could predict moderate/severe CIRR except for IMCs root development stage. IMC in a later stage of root development seems to be associated with a higher risk of moderate/severe CIRR. Further studies on the impact of CBCT for treatment planning of IMCs are warranted.
