# Supplementary material for: Radiographic features in 2D imaging as predictors for justified CBCT examinations of canine-induced root resorption
Source: Dentomaxillofac Radiol. 2022 Jan 1;51(1):20210165. doi: 10.1259/dmfr.20210165 (PMC8693324; doi:10.1259/dmfr.20210165)
Supplement: Supplementary Material 1. [file dmfr.20210165.suppl-01.docx]

**ONLINE SUPPLEMENTARY MATERIAL - METHODS**

Assessment of root resorption based on 2D radiographs and CBCT (gold standard)

- 1. Presence of resorption lacunae detected on 2D was recorded as (D) “Definitive”, (P) “Probably”, (PN) “Probably not” or (N) “No”, where D and P were assessed as “Yes”, and PN and N as “No”.
  2. Position of the root resorption was registered as (1) apical third, (2) middle third, (3) cervical third or (4) a combination, as evaluated in both 2D and CBCT volumes.
  3. Depth of the resorption lacunae evaluated in reformatted CBCT volumes were in its extent towards the pulp registered as in the (a) cementum layer, (b) outer half of the dentine, (c) inner half of the dentine without pulp involvement or (d) inner half of the dentine with pulp involvement.

Assessment of possible predictors for CIRR on PAN:

1. Anomaly in the same quadrant. This could be a missing or peg-shaped lateral incisor, YES or NO.
2. Distal tipping of the lateral incisor, YES or NO.
3. CCT position
4. Free of overlapping
5. Overlapping with lateral incisor on the apical third
6. Overlapping with lateral incisor on the middle third
7. Overlapping with lateral incisor on the cervical third or inferior of cervical third
8. Overlapping with lateral and central incisor on the cervical third or inferior of the cervical third
   1. Root development of the IMC
   2. Root shorter than crown
   3. Root longer than crown
   4. Close to full root length with open apex
   5. Full development with closed apex
   6. Inclination of IMCs, measured in degrees, in relation to midline
   7. Inclination of IMCs, measured in degrees, in relation to long axis of lateral incisor
   8. Inclination of IMCs, measured in degrees, in relation to occlusal plane
